# A Rare Presentation of Hand, Foot, and Mouth Disease During Pregnancy

**DOI:** 10.7759/cureus.28401

**Published:** 2022-08-25

**Authors:** Mohammad R Alam, Nabin Rokaya, Smritee Mahat, Ashutosh Upadhyaya, Pranjal Rokaya

**Affiliations:** 1 Internal Medicine, Arghakhanchi District Hospital, Arghakhanchi, NPL; 2 General Practice, Humla District Hospital, Simikot, NPL; 3 Pathology, National Trauma Center, National Academy of Medical Sciences, Kathmandu, NPL; 4 Internal Medicine, Evergreen Hospital Pvt. Ltd, Parasi, NPL; 5 Surgery, KIST Medical College, Lalitpur, NPL

**Keywords:** coxsackievirus, hfmd, coxsackie, enterovirus, obstetric, stillbirth

## Abstract

This case report is about an 18-year-old primigravida diagnosed with hand, foot, and mouth disease (HFMD) at 34 weeks of gestation. She had classic rashes on her hands and mouth but was otherwise healthy. The baby was delivered at 39 weeks via spontaneous normal vaginal delivery and was well after delivery. We provide a brief literature review on HFMD in pregnancy and a brief discussion on complications caused by the causative organisms.

## Introduction

Hand, foot, and mouth disease (HFMD) is a contagious viral illness that primarily affects children younger than five years of age and has seasonal outbreaks across East and Southeast Asia [1]. However, there have been reports of cases in populations other than the pediatric group, even though they are uncommon. It can sometimes lead to an outbreak that raises public health concerns.

HFMD is a highly contagious viral disease caused by a group of coxsackieviruses and enteroviruses. It is most commonly caused by coxsackievirus (CV) A16 and enterovirus (EV) A71 [2]. Typically, it begins with fever, malaise, and blisters on the hands and feet, which then spreads to other parts of the body. There are many ways through which viruses might spread, including direct contact and the spread of airborne droplets. Although in most cases, it resolves spontaneously within seven to 10 days, there are some reports of severe cases, such as progression to meningitis and encephalitis. Early rapid intervention is required to mitigate the disease progression in these cases [3].

It is relatively common for EVs to be passed from mother to baby (30-50%) [4]. However, their transmission rate through the placenta and the correlation between fetal infection and developmental outcomes are still unclear. In the literature, it has been suggested that when women are infected during pregnancy, the virus passes through the placenta and infects the fetus, consequently contributing to many unexplained cases of fetal and neonatal loss [5,6].

It is critical to document the effects of gestational HFMD infection to assess fetal and maternal risks and design appropriate clinical care for affected pregnant women. Most of the time, the clinical presentation is used to make a diagnosis. A pregnant woman presented to our hospital with an unusual presentation of HFMD. Therefore, we decided to report the effects of HFMD on obstetrical and neonatal outcomes.

## Case presentation

**Figure 1:**
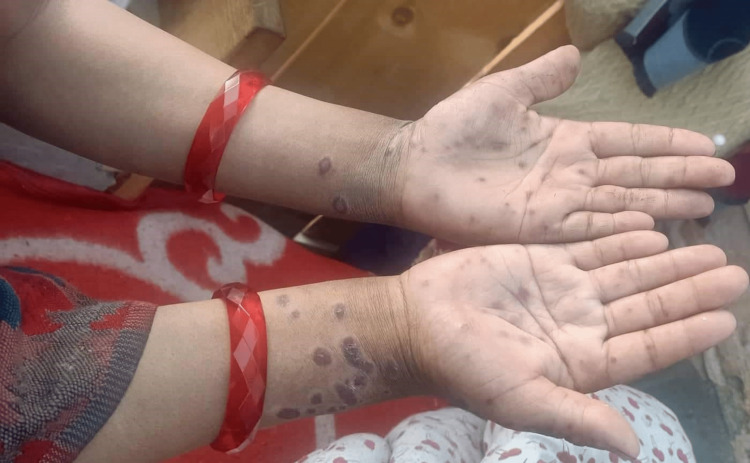
Lesions on the palm of the patient

**Figure 2:**
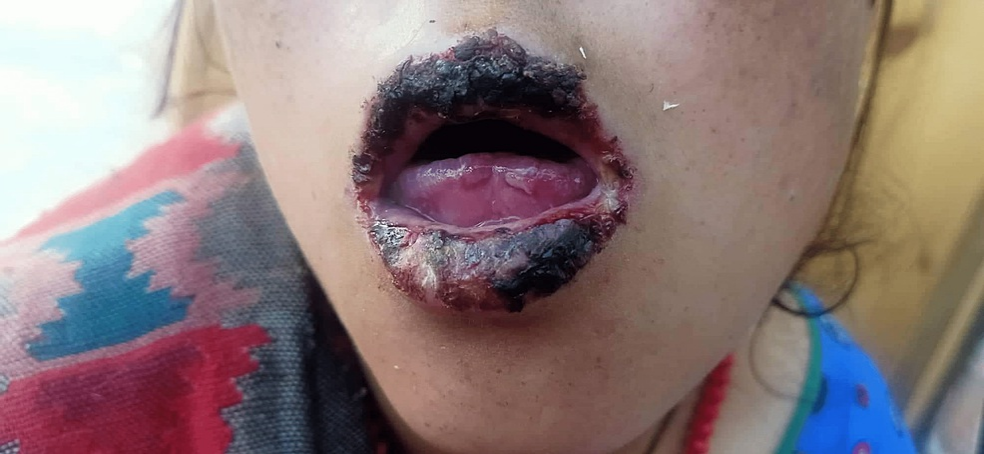
Lesions in the mouth of the patient

An 18-year-old primigravida at 34 weeks of gestation presented with a complaint of rashes on her hands and mouth. She had a history of two to three days of fever, malaise, and lethargy, followed by rashes. On physical examination, healed erythematous non-pruritic rashes were present on the ventral aspect of the forearms, palms (Figure 1), mouth (Figure 2), buttock, and upper thighs. However, no rashes were seen on the soles. She had been taking iron tablets and calcium supplements since her second trimester of pregnancy. There was no history of intake of other medication.

Routine laboratory study (Table 1) and obstetric ultrasonography were performed upon her admission. The routine blood and serological report and an obstetric scan revealed no abnormality in the mother and infant. We considered four differential diagnoses: HFMD, herpes, secondary syphilis, and adverse drug reactions. We were able to exclude syphilis as a possibility due to the fact that the syphilis serology result from the first trimester and the most recent report were both negative. Due to the fact that rashes on the palm are not typically associated with herpes, this diagnosis was immediately eliminated. Additionally, the absence of a recent history of drug use eliminated the possibility of an adverse drug reaction. As a result of the patient's history, clinical presentation, and serology report, HFMD was determined to be the primary factor in this case. The patient was managed with antibiotics, cefixime tablet 200 mg two times/day by mouth for seven days (to prevent secondary infection), analgesics, steroids, and supervision for five days in the hospital (in-patient). Her condition improved insidiously in four days with no alarming complaints like dysphagia, disorientation, headache, blurry vision, and preterm labor. Hence, she was discharged and asked to follow up as required.

**Table 1:** Blood work ESR: erythrocyte sedimentation rate; CRP: C-reactive protein; HIV: human immunodeficiency virus; VDRL: venereal disease research laboratory.

Laboratory parameter	Laboratory result	Reference
Hemoglobin	14.7 g/dL	12.0-16.0 g/dL
Total leukocyte count	5100/mm^3^	4500-11,000/mm^3^
Segmented neutrophil	41%	54%-62%
Lymphocyte	55%	25%-33%
Eosinophil	1%	1%-3%
Basophil	0%	0%-0.75%
Monocyte	3%	3%-7%
Platelet count	478,000/mm^3^	150,000-400,000/mm^3^
Serum creatinine	0.6 mg/dL	0.6-1.2 mg/dL
Blood urea	25.2 mg/dL	7-18 mg/dL
Random blood sugar	110 mg/dL	<140 mg/dL
Sodium	135 mmol/L	136-146 mEq/L
Potassium	4.3 mmol/L	3.5-5.0 mEq/L
ESR	17 mm/hr	<20mm/hr
CRP	18.8 mg/dL	
HIV	Non-reactive	
VDRL	Non-reactive	

She was again admitted with labor pain at 39 weeks of gestation. She vaginally delivered a healthy infant with an APGAR (appearance, pulse, grimace, activity, and respiration) score of 9, without any obstetric or neonatal complications.

## Discussion

Recently, HFMD infection rates have increased among adults despite being more common among children [7]. Immunosuppressed adults, such as pregnant mothers, are more susceptible to infection during the late stages of pregnancy when their immune systems are suppressed to avoid fetal rejection [8]. Although we do not yet know the effective rate of EV vertical transmission, several epidemiological, serological, and virological studies indicate that it is possible [9]. The literature indicates that EV transmission can occur antenatally or perinatally [10], resulting in severe neonatal infections if acquired shortly before delivery. However, few studies have examined the outcome of infection during early gestation [11,12].

There are few reports on EV infections during pregnancy due to their low prevalence in the population. According to Khediri et al., EV infection during pregnancy can cause miscarriage and fetal death [13]. It was reported by Euscher et al. that placental infection with CV could result in severe respiratory failure and central nervous system complications in newborns [14]. However, type 1 diabetes was reported in a study in 2011 [15].

Listed in Table 2 are some of the most relevant studies that have reported stillbirth, meningoencephalitis, and neonatal sepsis among neonates born to mothers diagnosed with HFMD during their second half of pregnancy. However, no obstetric or neonatal complications were seen in the above-mentioned cases. The evidence-based relationship between HFMD and its obstetric and neonatal complications is still an unanswered question that will presumably be addressed in future studies.

**Table 2:** Examples of neonatal complications due to enterovirus infections acquired during the second half of pregnancy

Year	Author	Neonatal complications
2002	Bauer et al. [16]	Neonatal disseminated intravascular coagulation
2006	Cheng et al. [17]	Neonatal intracranial hemorrhage
2004	Satosar et al. [18]	Neonatal death
2015	Yu et al. [19]	Stillbirth
1984	Laura et al. [20]	Meningoencephalitis

## Conclusions

The available literature indicates that there are relatively few reports of serious adverse effects on a fetus due to maternal HFMD. It could be concluded from the presentation of this case that fetal outcomes can be normal. However, since HFMD has been associated with severe complications, it would be prudent to monitor patients more closely and perhaps consider expediting delivery to prevent these complications. This topic has not been thoroughly studied, but researchers may find it relevant and valuable enough to explore the evidence-based association between HFMD and pregnancy outcomes in future studies.
